# Global Non-Communicable Diseases—The Nutrition Conundrum

**DOI:** 10.3389/fpubh.2018.00009

**Published:** 2018-01-29

**Authors:** Shweta Khandelwal, Anura Kurpad, K. M. Venkat Narayan

**Affiliations:** ^1^Public Health Foundation of India, Delhi, India; ^2^St John’s Research Institute, Bengaluru, India; ^3^Emory University, Atlanta, GA, United States

**Keywords:** nutrition epidemiology, bias, methods, life-course epidemiology, public health

Poor diets reduce our productivity and increase premature morbidity and mortality (1). Recent estimates suggest that ~45% of cardio-metabolic deaths in 2012 (318,000/702,000) were “associated with” top 10 dietary factors: fruits, vegetables, nuts/seeds, whole grains, unprocessed red meats, processed meats, sugar-sweetened beverages, polyunsaturated fats, seafood omega-3 fats, and sodium (2). Moreover, the health consequences of poor nutrition may be evident across the life-course and have intergenerational impacts (3–6). For example: pooled analyses from birth cohorts across several countries show that women with poor nutritional status give birth to babies which are disadvantaged in terms attaining their full cognitive potential and also have higher risk of developing chronic diseases in adult life (7–10). Thus, the scientific community has enthusiastically been pursuing several kinds of nutritional interventions across life span (in particular during early *in utero* life) to improve birth outcomes. However, nutrition science (especially nutritional epidemiology) poses particular challenges, for both science and communication of findings to the public.

As our knowledge regarding nutrition advances, some long accepted theories are challenged (11, 12). For example, the much celebrated Diet-heart hypothesis by Prof Ancel Keys is now being questioned in the research community. Researchers have argued that the Ancel Keys theory that more saturated fat causes higher CVD was promoted and accepted by the research and policy communities in absence of any other data and stringent cross-examination. This is currently being contested by several groups that speculate/suggest simple sugars are the bigger demon in nutrition epidemiology (13).

The purpose of this piece is to try and unpack some of the methodological challenges and obstacles that give rise to the confusion and contradictions in the evolving field of nutrition epidemiology. Below we describe some of the key challenges that underlie some of the contradictory findings and that need to be addressed to move the field forward:

1. Single vs. whole foods: Nutrition epidemiology is one of the most challenging types of research largely because everything in food can be split to indefinite micro levels. Natural foods (or their fractions) have repeatedly shown larger benefits than isolated single nutrients or fad diets. However, this must be viewed in the context of today’s environmental pollution and widespread use of pesticides and forbidden chemicals. The whole foods and natural products, therefore, may not exhibit all benefits as per our expectations. In all likelihood the interactions and effect modifications between constituents of food, various fractions, and sub fractions all exist and determine final impact. Therefore, two plus two does not yield four in nutrition studies as a lot of other factors modify outcomes. Nutrients alter epigenetic processes, and thus, it is important to pay attention to disease epidemiology holistically (14).
2. Plethora of confounders: Piece meal vs. holistic vision leads to varying and often erroneous interpretations. Multiple factors determine our intakes including but not limited to biology, physiology, demography, economy, religion, society, and lifestyle. How can we deduce the health impact of exact intakes in light of so many confounders? Even if we did, how much is bioavailable, of that how much gets metabolized and how? Advanced tools, improved methods and diagnostics unravel more and more layers of complexity to be unpacked and interpreted. The role of exercise, stress, our daily routines, home cooked vs. eating out, etc., plays a major role too in disease epidemiology.
3. Turning research findings into robust public health policy: for most people, the two important inter-related pieces of the puzzle are robust nutrition epidemiology leading to improved public health and nutrition. Dietary data collection approaches are largely limited to diet recalls and food frequency questionnaires. These methods, if not conducted skillfully, are at a risk of providing misleading data owing to measurement error issues (15). Furthermore, given the complexity of the issue and potential influence of non-research actors (e.g., industry), it may be difficult to meet these expectations, particularly in the context of a single study. Many factors determine the relevance of findings from a single (usually small) study, including the attention it garnishes in the media and public conscience. Whenever findings are repeated/cited/shown too often, we as humans tend to believe it. Our interpretation or discussion gets limited to what is published and largely excludes local context and experiential wisdom.
4. Commercial vs. public health interest—little knowledge is a dangerous thing! In the field of nutrition, research industry frequently has played a role in promoting confusion to their own advantage (16, 17). Lack of stringent laws and policies allows industry to frequently tweak the findings as per their whims and fancies. Promotion of flawed industry sponsored research further add to the confusion, as the general public may not have the tools to differentiate such research from research lacking such obvious conflicts of interest. Unfortunately, the industry controls advertising, which is used to reinforce public belief in the benefits of products laden with isolated fractions or nutrients regardless of whether they have any true health benefit. Furthermore, even inter-departmental and/or inter-ministerial harmonization of government policies and messages are critical. We view this often in agriculture and nutrition—goods being given subsidies to promote production may not be the best in terms of health benefits (18). Most of our decision makers and their real advisors are far from being researchers or scientists (a community believed to have conflicts within their own silos); so how do we as researchers more effectively communicate our findings, including their limitations?

Given the above challenges, the following strategies may be helpful in making the contribution from nutrition science more relevant to public health.

1. Telescope the transition—we must expand our horizon and think of preventive measures rather than curative ones. Multiple transitions viz nutrition, demographic, epidemiologic, etc., are occurring simultaneously and it is important to be ready in advance. We must think of potential public health issues lunging at us and start working at their solutions well in advance.
2. Build and strengthen capacity—the obvious is to invest in capacity to conduct high quality nutrition research studies, but we urge to equally pay emphasis on building skills of scientists to interpret and communicate scientific findings to masses and policy makers. Public health nutrition needs strong and effective communicators and policy advocates. The training component needs to be built taking all this into account.
3. Engage in interdisciplinary life-course approach to research—embrace a much more inclusive research space in real life settings with other domains contributing or explaining the findings more holistically. Studying vulnerable populations may be difficult but should be preferentially chosen by young doctoral and postdoctoral fellows. Community-based studies, realistic life-inspired locally relevant interventions, context-specific maternal child health research should be encouraged. The relevance of evolving fields like nutrigenetics and nutrigenomics, which integrate several domains such as biochemistry, genetics, and nutrition need to be promoted and appreciated (19, 20).
4. Provide quality mentorship—mentors are hard but not impossible to find. Opportunities for training experienced people and those progressing steadily should be created and nurtured.
5. Improve funding and infrastructure to encourage young people to enter this domain—most people get attracted to career streams based on remuneration, facilities available and future job security. It will be important to ensure all the above to attract good quality human resource wanting to choose this area of work.
6. Stress on collaboration—high-quality content driven collaborative thinking may yield better and more meaningful findings especially in the field of diet and nutrition.
7. Monitor and evaluate—it is very important to go back and assess what you find or want to replicate, scale up, etc. Monitoring and evaluation will loop back into strengthened methods and robust processes that yield valid and reproducible results.

## Author Contributions

SK wrote the manuscript. AK and KN provided critical feedback and helped improve the manuscript considerably.

## Conflict of Interest Statement

The authors declare that the research was conducted in the absence of any commercial or financial relationships that could be construed as a potential conflict of interest.
